# First Case Report of Deferiprone-Induced Anaphylactic Reaction

**DOI:** 10.7759/cureus.57847

**Published:** 2024-04-08

**Authors:** Naharuthai Bumrungratanayos, Songphol Tungjitviboonkun

**Affiliations:** 1 Internal Medicine, Faculty of Medicine, King Mongkut's Institute of Technology Ladkrabang, Bangkok, THA; 2 Internal Medicine, Sirindhorn Hospital, Bangkok, THA; 3 Medicine, Sirindhorn Hospital, Bangkok, THA

**Keywords:** anaphylactic, allergy, deferiprone, iron chelation, drug hypersensitivity

## Abstract

A 56-year-old Thai male, known for allergies to penicillin, sulfa, and lincosamide, presented with hyperferritinemia. Upon initiating deferiprone therapy, the patient experienced recurrent episodes of dyspnea, culminating in anaphylactic shock. Treatment included subcutaneous epinephrine, intravenous chlorpheniramine, and hydrocortisone, which led to symptom resolution. This case constitutes the first case report of deferiprone-associated anaphylactic reactions.

## Introduction

Iron is an essential component of hemoproteins, which include myoglobin, hemoglobin, and cytochrome p450. It plays important roles, including oxygen transportation and immunology modulation. Hence, maintaining physiological levels of iron is crucial. Iron deficiency can result in anemia and eventually metabolic failure while iron overload can result in cellular damage due to the generation of oxidative stress and free radicals [1].

Clinically, excess iron will be deposited in various organs throughout the body. Major organs affected by excess iron are the liver, pancreas, and heart. Excess iron deposited in the liver can result in chronic liver disease and cirrhosis and eventually lead to hepatocellular carcinoma. It is also able to damage the heart muscle, which can result in cardiomyopathy, arrhythmia, and heart failure. If excess iron is deposited in the pancreas, it can disturb its endocrine function, resulting in diabetes mellitus. Other than that, iron is also deposited in other organs such as the thyroid, joints, and brain. Thus, chelation therapy is one of the most important treatments that is needed concomitantly with blood transfusion to prevent complications from excess iron deposition [2,3].

Iron chelation therapy has become a major treatment that improves the clinical outcome of patients with iron overload conditions. The Food and Drug Administration (FDA) has approved three chelators in clinical practice today: deferoxamine (DFO), deferiprone (DFP), and deferasirox (DFX). The usage of iron chelation serves multiple purposes, such as balancing the iron levels during blood transfusion or emergency use in conditions that need immediate intervention, such as heart failure and multiple organ failure. Each kind of treatment requires dose adjustment depending on the clinical condition and iron levels. Choices used for each iron chelator depend on the cost of medication, route of treatment, period of treatment, and disease profiles [2].

## Case presentation

A 56-year-old Thai man with a history of allergies to penicillin, sulfa, and lincosamide presented with hyperferritinemia during a routine check-up. He did not have any prior history of blood disorders, blood transfusions, or familial occurrences of such conditions. His ferritin level was 900 µg/L (30-300 µg/L). Physical examination showed a normal liver and spleen size, with no signs of lymphadenopathy. His blood results did not indicate any issues with the liver, heart, pancreas, or thyroid. His hemoglobin typing was normal. After discussing options with the patient, he decided to start taking deferiprone at a dosage of 500 mg/day.

Fourteen days after beginning deferiprone, the patient visited the emergency department due to dyspnea and cough. Physical examination revealed a body temperature of 37.0°C, blood pressure of 118/74 mmHg, a pulse rate of 95 beats/min, a respiratory rate of 22/min, and oxygen saturation of 97% on room air. Chest examination showed minimal inspiratory rhonchi in both lung fields. He was diagnosed with acute bronchitis and treated with a salbutamol nebulizer every eight hours, azithromycin 500 mg/day for three days, and prednisolone 20 mg/day for three days. The patient stopped taking deferiprone during the three-day hospital stay.

One day after discharge, he resumed taking deferiprone and experienced chest tightness and dyspnea within 10 minutes. The patient was readmitted for two days due to acute bronchitis. Treatment included levofloxacin 750 mg/day and dexamethasone 4 mg intravenous every 12 hours. Deferiprone was discontinued, and his symptoms gradually improved.

Eight days after discharge, the patient ingested deferiprone again and suddenly developed dyspnea. He returned to the emergency department with a blood pressure of 90/40 mmHg, a pulse rate of 120 beats/min, and an oxygen saturation of 95% on room air. Chest examination revealed wheezing in both lung fields. The patient received eight points on the Naranjo adverse drug reaction probability scale, signifying "probable or likely diagnosis” of adverse drug reaction. He was diagnosed with anaphylactic shock due to deferiprone. Treatment included subcutaneous epinephrine (0.4 mL), intravenous chlorpheniramine (10 mg), and intravenous hydrocortisone (100 mg). His symptoms improved over the next two days. He was advised to discontinue deferiprone and continue monitoring his symptoms. Two months later, his ferritin level was 841.6 µg /L.

## Discussion

This case highlights a rare but critical adverse reaction to deferiprone, presenting as anaphylactic shock in a patient with a history of multiple drug allergies. It underscores the importance of vigilance and prompt management in suspected drug-induced allergic reactions.

Deferiprone

Deferiprone (DFP) is the only oral iron-chelating drug that has indications to treat patients with transfusional iron overload. DFP has been widely used due to the high activity of iron excretion. The effectiveness of DFP is not affected in patients with renal impairment and it is also non-renal toxic. Hence, it was approved by the FDA to be the first-line drug for the treatment of iron overload in pediatric and adult patients with sickle cell anemia or transfusion-dependent conditions [4,5]. The side effects of DFP that have been reported are agranulocytosis in 0.6% of patients, neutropenia in 6%, gastrointestinal symptoms in 6%, musculoskeletal and joint pain in 15%, and zinc deficiency in 1%. However, there had been no prior report of anaphylaxis with DFP use [5].

Drug hypersensitivity reported in another class of iron chelators

Although there has been no report of anaphylactic reaction with deferiprone before, there are several reports of hypersensitivity reactions after the use of other iron chelator drugs.

For deferasirox, skin reactions are notable adverse reactions. In contrast, deferoxamine has been associated with anaphylactic reactions. The management of this adverse drug reaction typically follows established standard guidelines. However, in some cases, management approaches like desensitization have been utilized to facilitate ongoing treatment (Table 1).

**Table 1:** Drug hypersensitivity reported in deferoxamine and deferasirox

Year	Patient age	Drug used	Clinical manifestation	Author
2015	22	Deferasirox	Urticarial rash	Sharma et al. [6]
2013	85	Deferasirox	Maculopapular rash	Ohshita et al. [7]
2020	50	Deferasirox	Generalized rash and swollen eyelids	Sompornrattanaphan et al. [8]
2014	11	Deferoxamine	Urticaria, wheezing, and gastrointestinal symptoms	Surapolchai et al. [9]
1995	6	Deferoxamine	Urticaria-angioedema	Patriarca et al. [10]
2006	10	Deferoxamine	Hypotension, tachycardia, pruritus, and urticaria	Gülen et al. [11]

For adverse reactions due to the administration of deferasirox, 10% of the cases report side effects as skin reactions. Sharma et al. reported a case of deferiprone-induced arthropathy and urticaria made better by antihistamines. However, hypersensitivity reaction confirmation was not done for this patient [6]. Another report from Ohshita et al. also reported skin reaction after administering deferasirox. After the dechallenge of this drug, the symptoms subsided. However, the rechallenge of the drug was not done in this patient [7]. Sompornrattanaphan et al. reported a case of deferasirox-induced type 1 hypersensitivity reaction. The patient developed a generalized rash and swollen eyelids after taking deferasirox. The therapy was not switched because the patient's iron excretion was unresponsive to other chelators. Thus, graded desensitization was done, and the patient became tolerant to the drug [8].

For adverse reactions due to the administration of deferoxamine, there are several reports on anaphylactic reactions and rechallenge therapy. Surapolchai et al. reported a case report of a 13-year-old Thai boy with thalassemia who underwent desensitization after an anaphylactic reaction from deferoxamine. The patient was having urticaria, wheezing, and gastrointestinal symptoms. The skin pricked test was positive in this patient. Type 1 hypersensitivity was confirmed. Later on, the patient was successfully desensitized to deferoxamine and rechallenged to take deferoxamine at the previous dose [9]. Patriarca et al. reported a case of a six-year-old child who was treated with deferoxamine. After initiation of the drug, the patient developed urticaria and angioedema. The hypersensitivity test was negative in this patient. The patient underwent deferoxamine desensitization for 21 days and tolerated deferoxamine at the previous dose (400 mg/day, subcutaneously) [10]. Gülen et al*.* reported a case of a 10-year-old girl with a systemic allergic reaction after being administered deferoxamine. This patient presented with hypotension, tachycardia, pruritus, and urticaria after drug administration. The type 1 hypersensitivity test was negative in this patient. However, an intradermal test to deferoxamine was positive in this patient. The patient was hospitalized and underwent desensitization for 17 cycles. Later on, the patient was challenged with the drug and continued the drug at the same dose [11]. It is believed that one of the mechanisms behind the allergic reaction from deferoxamine arose from the local effect on mast cells and not from an immunological reaction. Lombardo et al. reported that after switching the route of administration of deferoxamine in patients who reported hypersensitivity from standard subcutaneous therapy, four of the patients in the study did not report any local and systemic reaction from deferoxamine during one year of therapy. The treatment outcome also showed that deferoxamine IV also significantly improved patients' clinical status and drastically decreased iron stores and ferritin levels [12]. However, there is no study or report on potential substances present in iron-chelating agents that may precipitate adverse drug reactions.

## Conclusions

Deferiprone-associated anaphylactic reactions, while uncommon, necessitate vigilance and immediate action. Patients with a history of drug allergies may be at increased risk, and careful consideration should be given before initiating deferiprone therapy.
